# The effect of antenatal education in small classes on obstetric and psycho-social outcomes: a systematic review and meta-analysis protocol

**DOI:** 10.1186/2046-4053-3-12

**Published:** 2014-02-13

**Authors:** Carina Sjöberg Brixval, Solveig Forberg Axelsen, Stig Krøger Andersen, Pernille Due, Vibeke Koushede

**Affiliations:** 1 National Institute of Public Health, University of Southern Denmark, Copenhagen C, Denmark

**Keywords:** Antenatal education classes, Obstetric, Labor, Birth, Parenting, Parenthood, Psycho-social, Stress, Postnatal depression

## Abstract

**Background:**

The aims of antenatal education contain both outcomes related to pregnancy, birth and parenthood. Both content and methods of antenatal education have changed over time without evidence of effects on relevant outcomes. The effect of antenatal education in groups, with participation of a small number of participants, may differ from the effect of other forms of antenatal education. The latest Cochrane review, assessed as up-to-date in 2007, concluded that the effect of antenatal education for childbirth or parenthood or both remains largely unknown. This systematic review and meta-analysis aims to assess the effects of antenatal education in small groups on obstetric as well as psycho-social outcomes.

**Methods/design:**

Eligible studies include individually randomized as well as cluster-randomized trials irrespective of language, publication year, publication type, and publication status. Only interventions carried out in the Western world will be considered in this review. We will search the databases Medline, EMBASE, CENTRAL, CINAHL, Web of Science, and PsycINFO using relevant search terms. Two independent review authors will extract data and assess risk of bias. Results will be presented as structured summaries of the included trials. A meta-analysis will be conducted. We will assess heterogeneity by using both the Chi-squared test and the I-squared statistic, and conduct subgroup analysis separately for various intervention types.

**Discussion:**

In healthcare systems with limited resources evidence of the effectiveness of services provided is important for decision making, and there is a need for policy makers to implement changes in healthcare systems based on scientific evidence. The effectiveness of antenatal education in small classes is still questioned. Therefore an up-to-date systematic review is needed.

This systematic review protocol was registered within the International Prospective Register of Systematic Reviews (PROSPERO) as number CRD42013004319.

## Background

Antenatal education is offered to pregnant women in most high-income countries, more recently also to expecting fathers. Antenatal education has the overall aim of providing expecting parents with strategies for dealing with pregnancy, childbirth and parenthood [1]. More specific aims include influencing health behavior, increasing confidence in women’s ability to give birth, informing about pain relief, and promoting breastfeeding.

Antenatal education has been sensitive to opinions and trends, and has undergone marked changes over time. In some periods the focus has mainly been on maternal exercise and relaxation techniques, in other periods on antenatal education in small classes with group discussions, and in others again on lectures in large auditoriums with information on childbirth and breastfeeding. Likewise, the number of sessions has also changed over time due to financial and structural changes in the healthcare sector [2]. All these changes have occurred without sound evidence of the effect of antenatal education on outcomes relevant to healthcare providers as well as expecting parents [3].

The current evidence points to the importance of interacting with fellow learners and the learning environment in order to obtain new competencies [4]. In antenatal education classes that have a small number of participants it may be possible to create an environment which enables expecting parents to discuss feelings and concerns. Furthermore, it may enhance their awareness of own resources and provide them with problem-solving strategies that enhance important competencies to cope with birth and parenthood [5]. However, this approach has not been subjected to thorough scrutiny.

A previous systematic review by Gagnon and Sandall [3] investigated the effect of structured antenatal education either to individuals or groups on a range of outcomes both related to the birth process and parenthood and concluded that the effect of general antenatal education for childbirth or parenthood or both remains largely unknown [3]. However, since then more randomized trials have been conducted and the results from these trials might alter this conclusion. An updated review is therefore due.

In healthcare systems with limited resources evidence of the effectiveness of services provided is important for decision making, and there is a need for policy makers to implement changes in healthcare systems based on scientific evidence [6]. An up-to-date systematic review is needed in order to raise evidence for the effectiveness of antenatal education in small classes compared to no or other forms of education. The aims of antenatal education are numerous and various and therefore the purpose of our systematic review will be to assess the effects of antenatal education in small classes on various outcomes related to obstetric as well as psycho-social factors. Therefore, the specific research question is:

In expecting parents in a Western setting: What are the effects of antenatal education in small classes on obstetric and psycho-social outcomes compared to no intervention, treatment as usual, or other types of educational programs?

## Methods and design

In accordance with the guidelines, this systematic review protocol was registered within the International Prospective Register of Systematic Reviews (PROSPERO) on 11 April 2013 (registration number CRD42013004319).

### Types of studies and participants

Eligible studies will include individually randomized trials and cluster-randomized trials irrespective of language, publication year, publication type, and publication status to assess the effect of antenatal education in small classes.

Preparation for birth and parenthood are very dependent on culture and contextual factors, such as the organization of the health system. Therefore we will exclude trials taking place in developing countries and only include studies conducted in Western countries. We define Western countries as OECD membership countries [7]. We will include studies of pregnant women and/or their partners that have provided their informed consent to participation in the given trial.

### Types of interventions

The experimental intervention must be delivered as an antenatal educational program offered by an educator to groups consisting of more than one individual/couple, related to the birth of an infant and/or preparation for parenthood.

The control intervention can be either no intervention, treatment as usual, or other types of educational programs. If two programs are compared, the most intensive will be considered the experimental intervention.

Co-interventions are allowed but must be equally delivered in both the experimental and control arm.

### Types of outcome measures

Results must include quantitative data for outcomes measured. Both outcomes assessed as self-reported, via registries, or reported by a health professional will be accepted. If outcomes are measured more than once during follow-up, we will use the measurement shortly after the intervention ends and at the longest follow-up to consider the intervention effect.

The primary outcomes are: proportion of participants who receive pain relief during labor; proportion of participants who receive obstetric interventions; mean endpoint score in scales assessing psychological and social adjustment to parenthood; and proportion of participants with symptoms of antenatal and postnatal depression and anxiety (measured as defined by the trial).

The secondary outcomes are: knowledge acquisition; maternal sense of control/active decision making during labor and birth; partner involvement at birth; breastfeeding success; infant care abilities; and social support (all measured as defined by the trial).

### Search methods for identification of studies

Extensive searches will be performed by an information specialist (SKA). Medline, EMBASE, CENTRAL, CINAHL, Web of Science, and PsycINFO will be searched. The terms will include the following: antenatal, prenatal, education, parent preparation. Searches will be limited to randomized trials. Search words will be adapted to each database. An example is given in Table 1.

**Table 1:** Medline search strategy, modified as needed for use in other databases

**Search**^**a**^	**Medline**
1	(antenatal OR prenatal OR pregnancy OR birth OR childbirth OR (labor OR labour) OR obstetric OR (delivery OR deliveries))
2	(education OR “parent education” OR preparation OR “parent preparation” OR “early intervention”)
3	1 AND 2

^a^Filters: Refined by *randomized controlled trial*, *humans*.

In addition, we will search for relevant trials in citations from identified papers and former reviews. There will be no language or publication date restriction. The searches will be re-run just before the final analyses and further studies retrieved for inclusion.

### Selection of studies and data extraction

We will conduct the selection of studies in two steps. First two of the three review authors (CSB, VK, SFA) will independently perform the initial screening of all titles and abstracts to determine eligibility of all studies identified through the literature search. Next two of the three review authors (CSB, VK, SFA) will independently assess the full papers identified as meeting inclusion criteria or where definite decision on exclusion could not be made from screening titles and abstracts. Any discrepancies between the two review authors will be resolved through consultation with a third review author (PD).

A PRISMA flow diagram of progress will be completed for the selection process.

Data from the included papers will be extracted to summary tables containing information on: population, study design, interventions, theoretical framework, outcomes, type of effect analysis, results, and information for assessment of the risk of bias.

### Assessment of risk of bias in included studies

Two review authors (CSB, VK) will independently assess the included trials according to a predefined risk of bias scoring key [8] in order to determine the likely presence or absence of biases which might have affected the internal validity of the trials. Any discrepancies between the two review authors will be resolved through consultation with a third review author (PD).

The scoring key includes the following characteristics:

– Selection bias: randomization sequence generation and allocation concealment.

– Performance bias: assessment of blinding of participants, personnel, and outcome assessment.

– Attrition bias: assessment of systematic differences in withdrawal of study participants between the groups compared.

– Reporting bias: assessment of systematic differences between reported and unreported findings. It will be assessed whether a trial protocol exists and whether outcomes in the published trial have been reported in a pre-specified way.

– Other sources of bias: assessment of whether sample size and power calculations of the trial are based on the reported outcome.

First, each trial will be evaluated according to each of the above-mentioned bias domains as either 'low’, 'unclear’, or 'high risk of bias’. Second, the trials will be will be rated by an overall risk of bias. All trials rated as 'low risk of bias’ in all domains will be scored 'overall low risk of bias’. All other trials will be scored 'overall high risk of bias’.

### Data analysis

Structured summaries of the included trials will be presented, structured around type of intervention, intervention content, population characteristics and type of outcome. Intervention effects from the included trials will be calculated and presented as risk ratios (for dichotomous outcomes) or standardized mean differences (for continuous outcomes) with 95% confidence intervals and two-sided *P* values for each outcome.

We anticipate that there will be limited scope for meta-analysis because of the range of different outcomes reported from trials on antenatal care. However, where trials have used the same type of intervention and comparator, with the same outcome measure, we will pool the results using a random-effects meta-analysis, with standardized mean differences for continuous outcomes and risk ratios for dichotomous outcomes, and calculate 95% confidence intervals and two sided *P* values for each outcome. Outcomes measured by ordinal scales are analyzed according to the method presented in the included trial.

In studies where the effects of clustering have not been taken into account, we will adjust the standard deviations for the design effect. Heterogeneity will be assessed using both the Chi-squared test and the I-squared statistic. We will consider an I-squared value greater than 50% indicative of substantial heterogeneity. We will conduct sensitivity analyses based on study quality.

If the necessary data are available, subgroup analyses will be done separately for various intervention types: specific class content (for example, childbirth, parenting), size of classes in the intervention, number of antenatal education sessions, timing of classes, specific teaching approaches (for example, didactic, experiential), or effects in specific population groups (for example, socio-demographic factors, parity). Likewise, we will do subgroup analyses based on risk of bias; comparing effects of interventions with 'overall high risk of bias’ and interventions with 'overall low risk of bias’.

Trial sequential analysis will be done for significant results [9]. This analysis reduces the risk of type I errors, which may occur in meta-analysis due to the repeated testing of significance.

Statistical analyses will be based on intention-to-treat and calculated using the Cochrane statistical package, Review Manager (RevMan 2003).

## Discussion

This systematic review will assess the literature on the effect of antenatal education in small classes on both obstetric and psycho-social outcomes and compare with no or other forms of education. Since the aims of antenatal education are various, the present review will evaluate the effect on a broad range of outcomes in order to capture any relevant effect.

In 2007 a systematic review by Gagnon and Sandall was conducted [3] evaluating the effect of both individual and group antenatal education for childbirth or parenthood. They concluded that high-quality evidence was lacking, and that the effects of antenatal education are largely unknown. However, since 2007 more randomized trials have been conducted and results from these trials might alter this conclusion. The present systematic review will partly update the results from Gagnon and Sandall’s systematic review. We, however, will limit our focus to trials of antenatal education in small classes conducted in a Western setting.

Antenatal education is dependent on culture as well as organization of the healthcare system. Since the purpose of this review is to contribute to guidance of decision making in the Western world, only trials conducted in Western countries will be included in this systematic review. Comparing effects of antenatal education across very different healthcare systems may give a misleading view of the effects in a Western setting.

In many countries antenatal education have changed dramatically over time without letting evidence guide decisions for these changes. The results from this systematic review will help guide policy makers in making evidence-based decisions on the field of antenatal education.

## Competing interests

The authors declare that they have no competing interests.

## Authors’ contributions

CSB, VK, SFA, and PD developed the design of the protocol, drafted the manuscript, and will participate in extracting data and interpreting the results. SKA has developed the search strategy and will perform the literature search. All authors have read and approved the manuscript.

## Authors’ information

CSB: Master of Science in Public Health, PhD student on a large randomized trial (the NEWBORN trial) evaluating the effect of a structured antenatal education program.

VK: Midwife, MPH, PhD; principal investigator of the NEWBORN trial.

SFA: Pharmaconomist, Exam.pharm.cons, MPH; research assistant on the NEWBORN trial.

SKA: Information specialist; has expertise in literature searching.

PD: Professor, dr.med.Sci.; workpackage-chair of Child Intervention Research as part of The Centre of Intervention Research and research director of the research program for Child and Adolescent Health at The National Institute of Public Health, University of Southern Denmark.
